# DNA and Protein Co-Immunization Improves the Magnitude and Longevity of Humoral Immune Responses in Macaques

**DOI:** 10.1371/journal.pone.0091550

**Published:** 2014-03-13

**Authors:** Rashmi Jalah, Viraj Kulkarni, Vainav Patel, Margherita Rosati, Candido Alicea, Jenifer Bear, Lei Yu, Yongjun Guan, Xiaoying Shen, Georgia D. Tomaras, Celia LaBranche, David C. Montefiori, Rajasekhar Prattipati, Abraham Pinter, Julian Bess, Jeffrey D. Lifson, Steven G. Reed, Niranjan Y. Sardesai, David J. Venzon, Antonio Valentin, George N. Pavlakis, Barbara K. Felber

**Affiliations:** 1 Human Retrovirus Pathogenesis Section, National Cancer Institute, Frederick, Maryland, United States of America; 2 Human Retrovirus Section, Vaccine Branch, Center for Cancer Research, National Cancer Institute, Frederick, Maryland, United States of America; 3 Institute of Human Virology, Department of Microbiology and Immunology, University of Maryland School of Medicine, Baltimore, Maryland, United States of America; 4 Duke Human Vaccine Institute and Departments of Surgery and Immunology, Molecular Genetics and Microbiology, Duke University, Durham, North Carolina, United States of America; 5 Department of Surgery, Duke University Medical Center, Durham, North Carolina, United States of America; 6 Public Health Research Institute, University of Medicine and Dentistry of New Jersey, Newark, New Jersey, United States of America; 7 AIDS and Cancer Virus Program, Leidos Biomedical Research, Inc., Frederick National Laboratory for Cancer Research, Frederick, Maryland, United States of America; 8 Infectious Disease Research Institute, Seattle, Washington, United States of America; 9 Inovio Pharmaceuticals, Inc., Blue Bell, Pennsylvania, United States of America; 10 Biostatistics and Data Management Section, Center for Cancer Research, National Cancer Institute, Bethesda, Maryland, United States of America; Emory University School of Medicine, United States of America

## Abstract

We tested the concept of combining DNA with protein to improve anti-HIV Env systemic and mucosal humoral immune responses. Rhesus macaques were vaccinated with DNA, DNA&protein co-immunization or DNA prime followed by protein boost, and the magnitude and mucosal dissemination of the antibody responses were monitored in both plasma and mucosal secretions. We achieved induction of robust humoral responses by optimized DNA vaccination delivered by *in vivo* electroporation. These responses were greatly increased upon administration of a protein boost. Importantly, a co-immunization regimen of DNA&protein injected in the same muscle at the same time induced the highest systemic binding and neutralizing antibodies to homologous or heterologous Env as well as the highest Env-specific IgG in saliva. Inclusion of protein in the vaccine resulted in more immunized animals with Env-specific IgG in rectal fluids. Inclusion of DNA in the vaccine significantly increased the longevity of systemic humoral immune responses, whereas protein immunization, either as the only vaccine component or as boost after DNA prime, was followed by a great decline of humoral immune responses overtime. We conclude that DNA&protein co-delivery in a simple vaccine regimen combines the strength of each vaccine component, resulting in improved magnitude, extended longevity and increased mucosal dissemination of the induced antibodies in immunized rhesus macaques.

## Introduction

DNA is a compelling vaccine vehicle because of its simplicity, scalability, and lack of immunity against the vector. The development of intramuscular (IM) DNA injection followed by *in vivo* electroporation (IM/EP) [1]–[6], brought a significant improvement in the efficiency of DNA delivery, especially to higher mammals like macaques and humans [7]–[11]. HIV/SIV DNA vaccine delivered by IM/EP leads to increased immune responses compared to those induced by conventional needle and syringe injection [9], [12]–[14]. The magnitude of the DNA induced immune responses could be further augmented by the inclusion of IL-12 DNA as adjuvant in mice and macaques [9], [15]–[21]. Importantly, in macaques, the combination of such an optimized SIV DNA vaccine regimen delivered by IM/EP led to higher cellular and humoral responses [9], [12], [20]–[24] with broader neutralizing activity [20]. Similar improvement in immunogenicity using HIV DNA and IL-12 DNA codelivered by IM/EP has been reported in humans in the recent HVTN 080 trial [7], [11], which resulted in the highest response rate in a phase I HIV vaccine trial and indicates that the macaque model has predictive value for human immunogenicity.

In the RV144 trial conducted in Thailand, the only HIV vaccine clinical trial to date that has shown a modest protective effect, the risk of contracting HIV-1 infection was found to inversely correlate with binding IgG antibodies to variable regions 1 and 2 (V1/V2) of the HIV-1 envelope [25]–[27]. These results emphasize the need of inducing potent Env-specific antibody responses with adequate specificity. To improve immunogenicity, some vaccine strategies use a prime/boost regimen with plasmid DNA followed by viral vector or protein boost [[28]–[31], and reviewed in [32]]. Alternatively, it was shown that HIV DNA&protein co-immunization elicited higher humoral immune responses in rabbits and mice compared to vaccination with either of the two individual components [33], [34]. This vaccine regimen also showed increased HIV Env antibody responses in a pilot study in macaques [34], and these responses were further augmented in the presence of an adjuvant. Importantly, we also reported that a vaccine combining SIVmac239 DNA and protein elicited systemic and mucosal SIVsmE660 binding antibody (bAb) responses, which correlated with slower virus acquisition upon SIVsmE660 challenge [24].

In the present work, we evaluated the magnitude, longevity and mucosal dissemination of humoral immune responses induced in macaques by three vaccine regimens including SIV DNA: DNA only, DNA&Protein co-immunization and DNA prime-protein boost. Our results indicate that the co-immunization protocol provides the most balanced and durable cellular and humoral immune responses.

## Results

### Co-immunization with DNA&Protein Elicits Highest Plasma Humoral Responses to SIV Env

**Figure 1:**
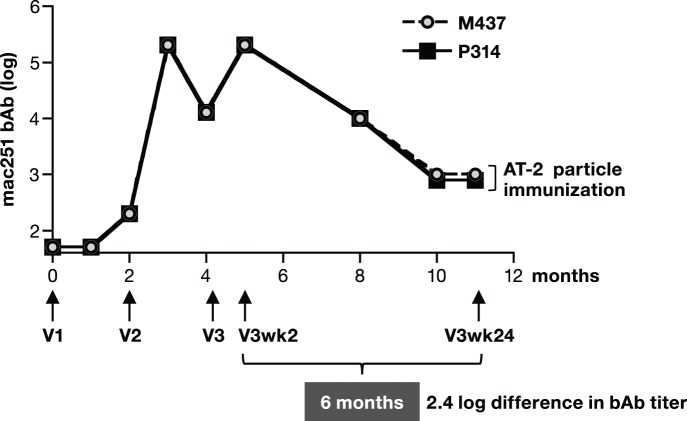
SIV Env antibody responses upon vaccination of macaques with AT-2 inactivated SIVmac239 virus particles. Endpoint bAb titers (log values) to SIVmac251 Env were measured from two macaques which were immunized 3 times with only SIV AT-2 particles. The protein was administered by subcutaneous and intramuscular injections into the same sites, immediately following sham DNA injection. The difference of log endpoint Env bAb titers between peak (data from 2 weeks post vaccination) and trough (6 months after V3) is shown.

**Figure 2:**
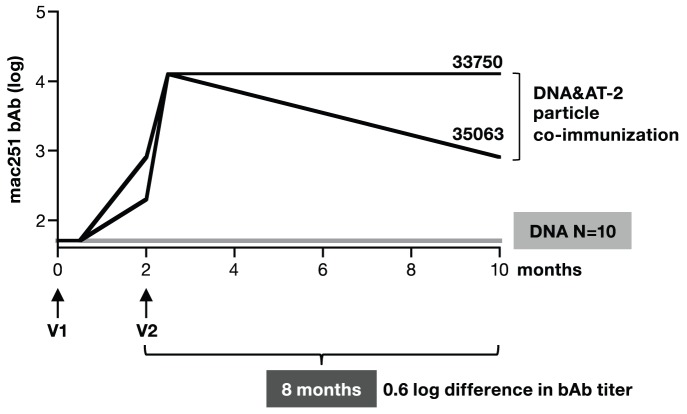
Comparison of humoral immune responses upon DNA and DNA&Protein co-immunization administered by IM injection. Macaques were immunized twice (V1, V2) at 0 and 2 months with SIV DNA only (N = 10) or co-immunized with DNA&Protein (N = 2) using SIV AT-2 particles as protein source. The DNA vaccine was administered by IM injection (needle/syringe). The protein was administered by subcutaneous and intramuscular injections into the same sites, immediately following the DNA injection. Endpoint bAb titers (in log) to SIVmac251 Env were measured. The endpoint values for the DNA only vaccinated animals were below the cut-off (1∶50) of the assay. Of note, the *env* DNA used in these animals [22] was different than the improved expression-optimized *env* DNA used in the subsequent studies presented in this work. In addition, we used a less efficient DNA delivery method, which explains the lower immunogencity of this DNA-only vaccine.

We performed a series of exploratory experiments intended to evaluate the durability of Env humoral responses in a small number of animals (**Figures 1** **,** **2**). We found that macaques vaccinated with SIV protein only in the form of AT-2 inactivated SIVmac239 viral particles (AT-2 SIV), developed robust Env-specific bAb responses after receiving a 2^nd^ and 3^rd^ vaccination (V2 and V3, respectively, **Figure 1**), but the responses declined rapidly after V2 and V3, with a decay of ∼2.4 log over the 6 months following V3. In contrast, we found that macaques co-immunized with a mixture of SIVmac239-derived DNAs administered by needle and syringe via the IM route together with AT-2 SIV particles developed Env bAb responses (**Figure 2**), which, interestingly, showed remarkable persistence over 8 months of follow-up. However, macaques immunized with plasmid DNA-only administered by needle and syringe via the IM route without electroporation failed to develop measurable antibody responses after 2 vaccinations (**Figure 2**) indicating that the co-delivery of protein was necessary to obtain the robust and lasting immunity.

**Figure 3:**
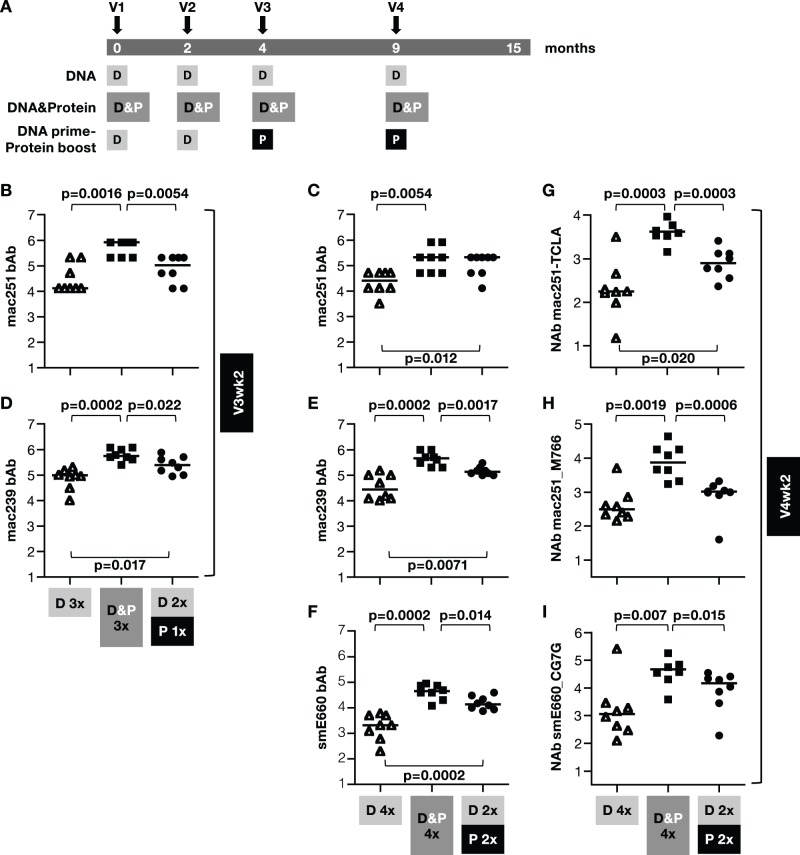
SIVmac239 DNA&Protein co-immunization induces highest bAb and NAb titers in plasma. (**A**) Three groups of macaques (N = 8) were immunized 4 times (V1 to V4) with SIVmac239 derived vaccines using regimens that included DNA (D) only, DNA & protein (P; AT-2 inactivated SIVmac239 viral particles) co-immunization, and DNA prime-protein (viral particles) boost. (**B–F**) Endpoint bAb titers (in log) were measured to SIVmac251 (**B, C**), SIVmac239 (**D, E**) at 2 weeks post V3 (**B, D**) and V4 (**C, E**), and the heterologous SIVsmE660 (**F**) Env at 2 weeks post V4. NAb titers were measured from individual plasma samples against SIVmac251-TCLA (**G**), the transmitted SIVmac251_M766 (**H**) and the heterologous SIVsmE660_CG7G (I) at 2 weeks post V4 (log values). Median values are indicated. P values using non-parametric two-tailed t-test (Mann-Whitney) are shown.

We expanded on these observations with a subsequent study, where we implemented several vaccine improvements, i.e. better *env* DNA plasmid, inclusion of rhesus IL-12 DNA as adjuvant, improved DNA vaccine delivery by IM injection followed by EP, and the inclusion of protein as a vaccine component. Three groups of Indian rhesus macaques (N = 8/group) were vaccinated 4 times (V1 to V4 at month 0, 2, 4, 9, respectively; **Figure 3A**) with a SIVmac239-derived vaccine consisting of DNA alone or DNA and AT-2 SIV either in a co-immunization regimen or in a classical prime-boost vaccination protocol after 2 DNA priming vaccinations as reported previously [24]. All groups received an expression-optimized DNA vaccine consisting of plasmids expressing different SIVmac239 genes delivered via IM/EP. Endpoint antibody titers against a panel of SIV Env proteins including SIVmac251, SIVmac239 and SIVsmE660_CG7V, were determined in individual plasma samples 2 weeks post V3 (**Figure 3B, 3D**) and post V4 (**Figure 3C, 3E–F, S** **1**). SIVsmE660_CG7V, differs by 20% from the SIVmac239 Env used as vaccine.

The DNA-only vaccine induced robust responses able to recognize a panel of different Env including SIVmac251 (**Figure 3B–C** **; S1A**), SIVmac239 (**Figure 3D–E**), and the heterologous SIVsmE660 (**Figure 3F, S** **1B**). Animals immunized with the vaccine regimen that included a protein boost at V3 and V4 had significantly higher bAb titers against SIVmac251, SIVmac239 and the heterologous SIVsmE660 compared to the DNA-only group, supporting the notion that a vaccine based on DNA as the only component may not induce maximal anti-Env humoral immune responses. In contrast, the co-immunization regimen induced the highest Env bAb levels against a panel of Env proteins tested (**Figure 3B–F** **; S1**).

We further examined whether the three vaccine regimens differ in their ability to induce neutralizing antibodies (NAb). The plasma samples were examined for their ability to neutralize a panel of SIVmac and SIVsmE660 pseudotyped viruses at 2 weeks post V4 (**Figure 3G–I**). We found that SIVmac239-derived DNA-only vaccination induced broadly NAb recognizing TCLA-SIVmac251 (**Figure 3G**), the transmitted SIVmac_M766 (**Figure 3F**) as well as the heterologous tier-1a-like SIVsmE660_CG7G (**Figure 3I**) Env. Significantly higher NAb titers developed using the vaccine protocols that included protein, with the co-immunization regimen inducing the highest NAb levels. Together, using different assays to measure bAb and NAb, our data demonstrate that the highest humoral responses were induced by the co-immunization regimen.

**Figure 4:**
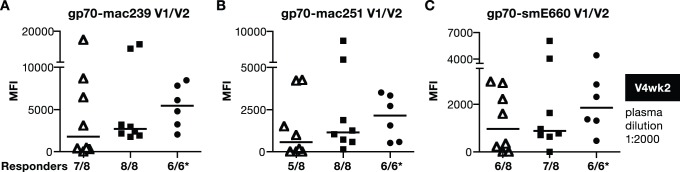
All vaccine regimens induce antibodies to V1/V2-scaffold antigens. V1/V2-specific antibody responses in individual plasma samples from the vaccinated macaques described in Figure 2 were measured at 2 weeks post V4 using SIV-BAMA. A panel of V1/V2-scaffold gp70 proteins derived from mac239 (**A**), mac251 (**B**) and smE660 (**C**) were tested. The responses were measured using a 1∶2000 dilution of the plasma. Median values are shown. Asterisk (*) denotes that samples from only 6 macaques were available from the DNA prime/protein boost group.

Since bAb to V1/V2 were identified to correlate with decreased risk of infection in the RV144 trial, we investigated the responses induced by these vaccine regimens for their ability to recognize a panel of V1/V2-scaffold gp70 recombinant proteins containing SIVmac or SIVsm V1–V2 regions at V4wk2 (**Figure 4**). Although the DNA-only vaccination regimen induced lower bAb responses, it is noteworthy that these antibodies were able to recognize epitopes present in V1/V2-scaffold regions of mac239 (**Figure 4A**), mac251 (**Figure 4B**) and the heterologous smE660 (**Figure 4C**). Despite the higher antibody titers obtained upon inclusion of protein in the vaccine regimen, no significant difference in the recognition of the V1–V2 regions was found among the groups. We concluded that addition of a protein component to the DNA vaccine did not alter V1-V2 recognition but significantly increased the magnitude of the humoral responses when compared to the DNA or the DNA prime-protein boost vaccines.

We further analyzed the cellular immune responses induced by these vaccine regimens (**Figure S2**). Comparison of DNA-only and the DNA&Protein co-immunization regimen upon 2 vaccinations (V2wk2) showed similar levels of Env-specific cellular responses (**Figure S2A**), despite differences in humoral responses. We noted an animal-to-animal heterogeneity in the induced cellular immunity as reported previously for out-bred macaques by us and others [22], [24]. Both vaccine regimens induced mainly Env-specific CD4^+^ T cell responses (Figure S2B). Thus, co-immunization with protein did not affect the magnitude or specificity of the cellular Env-specific responses. This conclusion is supported by additional studies comparing macaques vaccinated with DNA or co-immunized with DNA&Protein (unpublished). Comparing DNA only and the DNA&Protein co-immunization groups, we previously reported [24] a similar distribution of total SIV-specific memory T-cell responses with transitional and effector memory phenotypes as well as the induction of similar levels of SIV-specific granzyme B^+^ cytotoxic T cells. Upon additional vaccinations (V4wk2), similar levels of cell-mediated responses were found using the DNA and DNA&Protein co-immunization regimens (**Figure S2C**)**.** On the other hand, the DNA prime-protein boost group showed lower responses at V4wk2 (**Figure S2C**) as expected, because these responses were measured 5.5 months after the last DNA vaccination (vaccination 2 for this group). Thus, comparison of the immune responses induced after 4 vaccinations showed that the DNA prime-protein boost group has high humoral but low cellular responses, the DNA-only group showed the lowest humoral but high cellular responses, whereas the DNA&Protein co-immunization showed both high humoral as well as cellular immunity, combining the advantages of both vaccine modalities.

**Figure 5:**
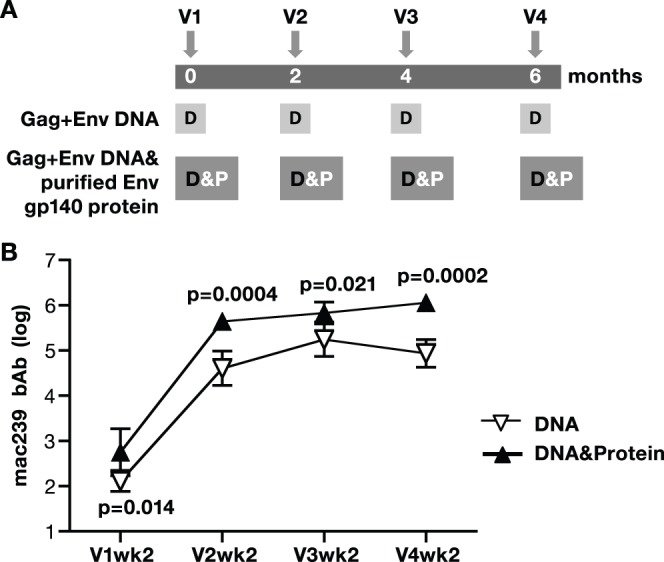
Env bAb titers in macaques immunized with DNA (D) or DNA together with purified SIV Env (D&P). (**A**) Outline of vaccination study. Macaques (N = 4/group) were immunized 4 times (V1 to V4) with SIVmac239 derived vaccines using protocols that included DNA only (D) and DNA&protein (P; SIV gp140 purified from human HEK293 cells) co-immunization. (**B**) Endpoint bAb titers (log values) to SIVmac239 Env were measured at 2 weeks after each vaccination. Mean and standard deviation (SD) are indicated. P values using repeated measures ANOVA are shown.

To exclude that the increased immunogenicity of the DNA&protein co-immunization regimen was due to an unspecific adjuvant effect of cellular proteins present in the SIV AT-2 particle preparation, we tested the co-immunization regimen using purified SIV gp140 Env protein produced in HEK293 cells (**Figure 5**). Macaques received a mixture of *gag* and *env* DNA alone or together with 100 µg gp140 Env protein adjuvanted in the synthetic EM-005 (GLA-SE) [35], a mixture of squalene and a TLR-4 agonist (**Figure 5A**). Analysis of humoral responses showed that inclusion of Env protein led to significantly higher endpoint Env bAb titers (**Figure 5B**) and this difference was maintained over the course of the 4 vaccinations. Although these data were obtained in the presence of adjuvant (EM-005), we previously reported a macaque pilot study where DNA&HIVgp120 protein co-immunization also induced higher humoral responses in the absence of adjuvant, but the inclusion of EM-005 further increased the humoral responses [34]. Thus, co-immunization regimens including SIV Env proteins (**Figure 5**) or unadjuvanted SIV AT-2-inactivated particles (**Figures 2** **,** **3** **, S1**) elicited significantly higher humoral responses compared to DNA alone.

Taken together, these data show that the robust broad antibody responses elicited by DNA only vaccination could be increased by vaccine regimens including either DNA as prime followed by protein boost or, most importantly, co-immunization of DNA&protein, a regimen that combines the benefit of both vaccine modalities and induces highest and broadest bAb and NAb responses.

### Inclusion of DNA in the Vaccine Protocol Increases the Longevity of Antibody Responses

**Figure 6:**
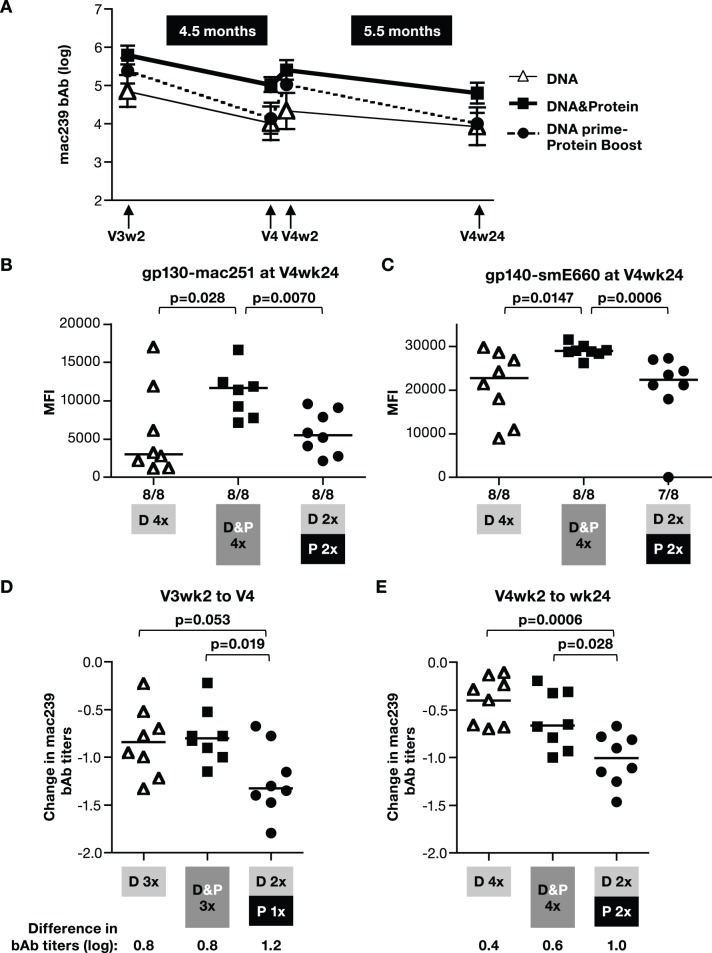
DNA&Protein co-immunization increases the longevity of humoral responses. (**A**) SIVmac239 Env endpoint bAb titers (in log) were determined for individual animals described in Figure 3 after V3 and V4. Mean and SD of the 3 groups are shown. (**B, C**) Persistence of systemic humoral responses at 24 weeks after the 4^th^ vaccination. bAb to SIVmac251 (**B**) and SIVsmE660 (**C**) Env were measured at V4week24 from the three groups of vaccinees. Individual plasma samples were analyzed at a 1∶80 dilution using SIV-BAMA. Bars represent the median values. The p values are given (non-parametric t-test Mann-Whitney). (**D, E**) Difference of log endpoint mac239 bAb titers between (**D**) V3wk2 and V4 and (**E**) V4wk2 and V4wk24 are shown using the measurements shown in panel A. Bars represent the mean values. The p values are given (non-parametric t-test Mann-Whitney).

We further monitored the humoral responses for 6 months after the last vaccination (**Figures 6A**). We noted that the macaques receiving the DNA&Protein co-immunization vaccine maintained the highest bAb levels (**Fig. 6B–C**). We examined differences in the longevity of the Env responses among the 3 vaccine groups (**Figure 6A**) by comparing the decline of the antibody titers during the 4.5 months between the V3wk2 and V4 and during the 5.5 months between V4wk2 and V4wk24 (**Figure 6D–E**). The bAb titers in the 2 groups immunized with DNA only or co-immunized with DNA&protein showed similar declines (∼0.8 log) overtime, whereas the group receiving DNA prime-protein boost showed the least persistent humoral immune responses, with a significantly greater decline of ∼1.2 log (**Figure 6D**). Similar observations were made over the 5.5-month period following the last vaccination (**Figure 6E**) with a decline of 0.4–0.6 log for the DNA only and DNA&protein groups, and a significantly greater decline of ∼1 log for the DNA prime-protein boost group. Thus, the responses induced by each protein boost were not maintained; these findings are reminiscent of the results obtained from the macaques vaccinated only with SIV AT-2 particles (**Figure 1**) that showed a decline of the bAb titers ∼2.4 log over the 6 months after the 3^rd^ vaccination.

**Figure 7:**
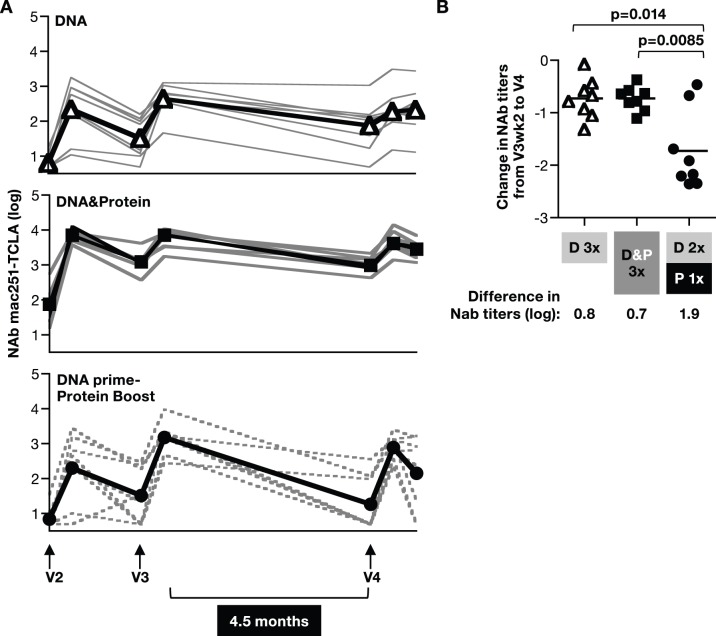
Persistence of NAb in plasma. (**A**) Log NAb titers against SIVmac251-TCLA were measured over time in individual plasma samples from the vaccinated macaques described in Figure 3. The black line denotes the median titers. (**B**) Difference of SIVmac251-TCLA titers measured in individual plasma samples between the 4.5 months of follow-up between V3wk2 and V4 (data from panel A). The mean difference in NAb titers (indicated by a bar) and the p values comparing the NAb titers at these time points from the respective groups are given (score test of the proportional hazards model accounting for censored differences).

Similar to the pattern observed for the bAb, the NAb titers to TCLA-mac251 Env in the DNA only and the DNA&protein groups were better maintained, whereas the group receiving the protein boost showed a sharp decline in the responses (**Figure 7A**). Analysis of NAb responses over the 4.5 months between V3wk2 and V4 also showed that DNA only and DNA&protein groups had similar declines of ∼0.7 log, whereas the animals in the DNA prime-protein boost group showed a significantly greater decline of ∼1.9 log (**Figure 7B**).

**Figure 8:**
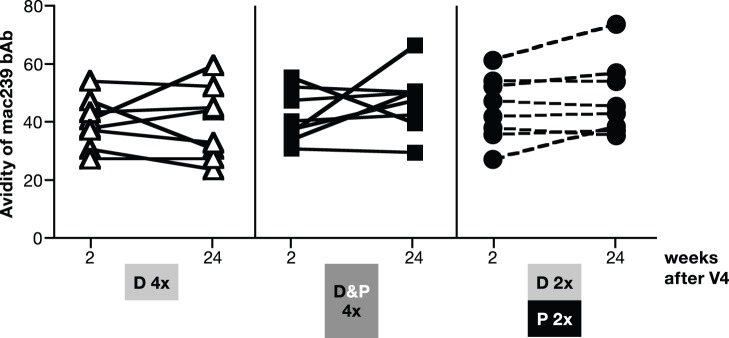
Persistence of SIVmac239 Ab avidity index. The avidity of anti-SIVmac239 Env bAb elicited in the 3 groups of macaques described in Figure 3 was measured at 2 and 24 weeks after the last vaccination (V4).

**Figure 9:**
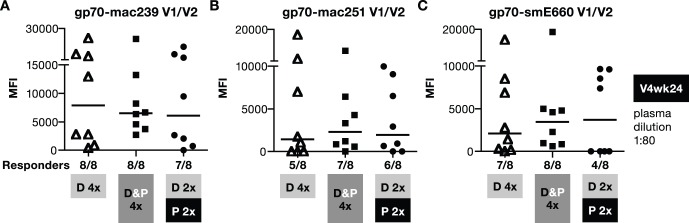
Induction of durable antibodies to V1/V2-scaffold antigens. V1/V2-specific antibody responses in individual plasma samples from the vaccinated macaques described in Figure 3 were measured at 24 weeks post V4 using SIV-BAMA. A panel of V1/V2-scaffold gp70 proteins derived from mac239 (**A**), mac251 (**B**) and smE660 (**C**) were tested as described in Figure 4. Note, the responses were measured using a 1∶80 dilution of the plasma due to the lower antibody levels at V4wk24. Therefore, the assay was performed using less diluted samples than that used for the analysis of the V1V2 responses at V4wk2 (**Figure 4**). Median values are shown.

Together, these data demonstrate that the DNA&Protein vaccine achieved both high magnitude of bAb and NAb and improved longevity of the vaccine-induced humoral responses. Despite the difference in the longevity of the humoral responses among the groups, the avidity to mac239 Env (**Figure 8**) did not show any difference among the vaccine groups (V2wk2) and remained unchanged during the 5.5-month follow-up (V4wk24). We further noted that the ability of the bAb to recognize a panel of V1/V2-scaffold gp70 proteins also remained in all groups, although to a lesser extent (**Figure 9A–C**).

**Figure 10:**
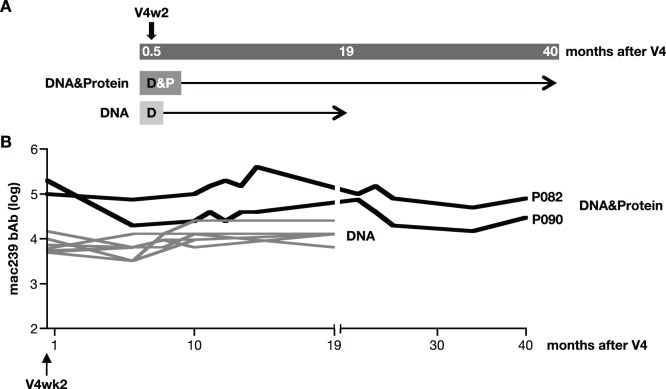
Durability of humoral immune responses upon DNA vaccination up to 40 months post the last vaccination. (**A**) Two groups of vaccinated macaques were monitored for the maintenance of bAb responses to SIVmac239 for several years after the last vaccination. Macaques P082 and P090 are from the DNA&Protein group (see Figure 3). The group of 8 DNA-only vaccinated macaques [20] received the same vaccine described in the DNA-only group described in Figure 3 but without IL-12 DNA as adjuvant. (**B**) Endpoint SIVmac239 Env bAb titers (in log) were measured from macaques P082 and P090 over 40 months after V4 and from the DNA-only group over 19 months after V4.

Further evidence of extended longevity of the antibody responses induced by vaccine regimens that contain DNA was obtained with two animals from the DNA&Protein co-immunization group (P082, P090), which showed persistence of the Env bAb levels for >3 years (**Figure 10**). Similarly, in another cohort of animals immunized with DNA only, sustained Env antibody responses were observed for >1.6 years (**Figure 10**). Together, these data support the notion that inclusion of DNA in the vaccine is critical to obtain long-lasting immunity, whereas the presence of protein in the vaccine leads to augmentation of the responses.

### DNA/Protein Vaccination Promotes Dissemination of SIV Env-specific IgG to Mucosal Surfaces

**Figure 11:**
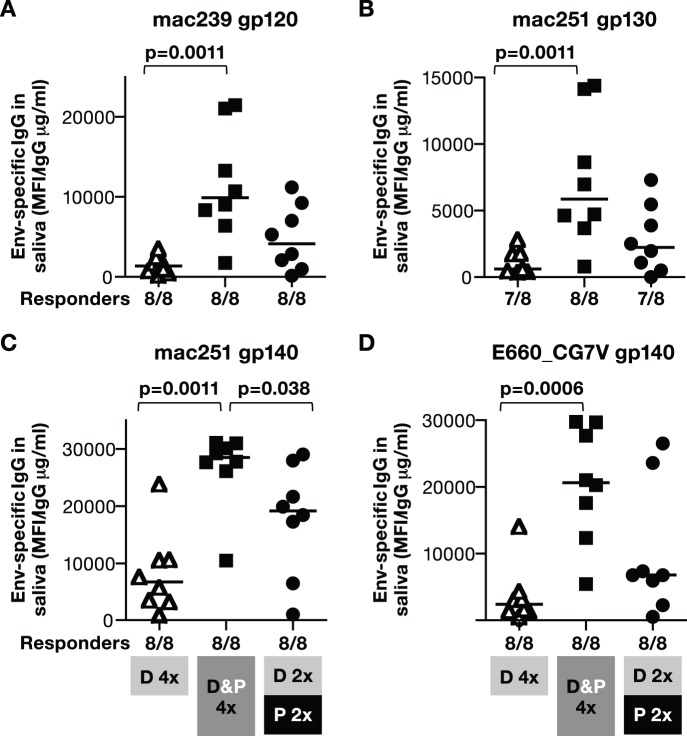
DNA&protein co-immunization induces higher SIV Env-specific IgG in saliva. SIV-specific IgG was measured in saliva of immunized macaques (from Figure 3) at 2 weeks post the last immunization (V4). The SIV bAb multiplex assay (SIV-BAMA) included SIVmac239 gp120 (**A**), SIVmac251 gp130 & gp140 (**B, C**), and the heterologous SIVsmE660 gp140 **(D**)**.** Median values are indicated. P values using non-parametric two-tailed t-test (Mann Whitney) are shown.

**Figure 12:**
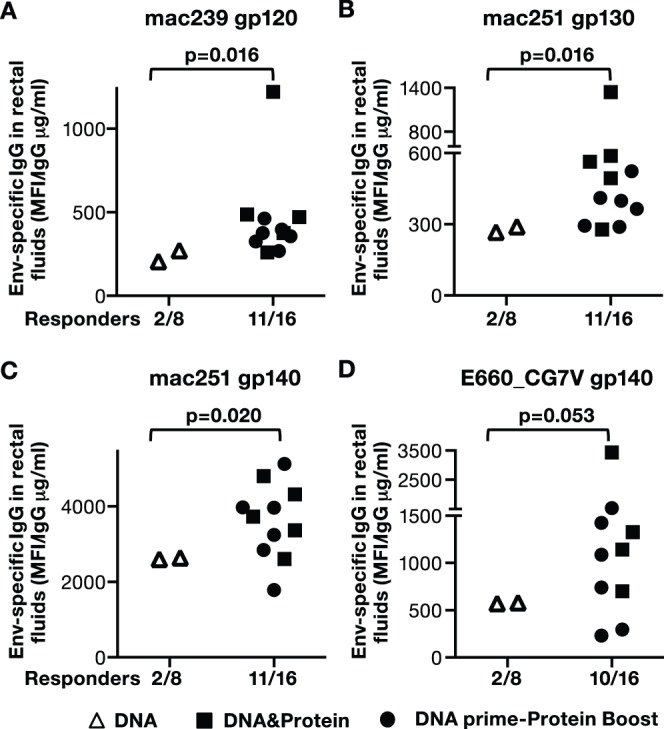
Dissemination of SIV Env-specific IgG to rectal mucosa upon IM/EP DNA vaccination. SIV-specific IgG were measured in rectal fluids of immunized macaques (from Figure 3) at 2 weeks post last immunization (V4). SIV bAb multiplex assay included SIVmac239 gp120 **(A**), SIVmac251 gp130 & gp140 **(B, C**), and SIVsmE660 gp140 **(D**). The frequency of the responders in the DNA only group was compared to the combination of regimens including protein. The DNA&Protein group had 5 of 8 responders, except for panel **D**, which had 4 of 8 responders. Animals scoring negative for rectal Ab are not shown. The DNA prime-protein boost group had 6 of 8 responders. P values using non-parametric two-tailed t-test (Mann Whitney) are shown.

We also examined whether the humoral responses induced upon IM delivery by the three vaccine regimens were able to disseminate to mucosal surfaces. SIV Env-specific IgG levels were determined 2 weeks after the last immunization (V4) in saliva (**Figure 11**) and rectal mucosa (**Figure 12**) of immunized macaques.

Analysis of saliva samples from individual macaques from the DNA only group showed the presence of low levels of responses to mac239 Env (**Figure 11A**), to different forms of SIVmac251 Env (**Figure 11B–C**) as well as to heterologous SIVsmE660 Env (**Figure 11D**). Importantly, the Ab levels were significantly higher in the DNA&Protein co-immunization group. We noted a broader range of responses in the prime-boost group, which is likely responsible for the lack of significance in comparison to the DNA only group. Thus, DNA&protein co-immunization regimen had the advantage of consistently eliciting significantly higher humoral responses in the saliva. We also noted strong correlations between the responses across the different Env antigens, especially in the DNA&protein and DNA prime-protein boost groups. Thus, these data suggest that responses to different Env proteins were measured reliably in individual animals using the SIV bAb antibody multiplex assay (SIV-BAMA).

We further analyzed rectal secretions for the presence of Env-specific IgG recognizing the panel of SIVmac and SIVsm Env proteins using SIV-BAMA (**Figure 12**). We found that the DNA-only group had few responders (2 of 8 macaques), whereas the groups that included protein in the vaccine showed a significantly higher response rate (4–6 of 8 macaques). Comparison of the responders (25%) in the DNA only group to the responders in the combined group that received protein (11 of 16 animals or 60% positive responders) indicated that inclusion of protein in the vaccine is critical to induce the levels of humoral responses required to efficiently disseminate into mucosal surfaces. Thus, the co-immunization regimen has been the most efficient in inducing significantly higher and persistent systemic humoral responses able to disseminate into mucosal compartments.

## Discussion

Understanding the quality of immune responses induced by different vaccine regimens is essential for improving the prospects of AIDS vaccines. To identify the most robust regimen, we compared vaccination protocols that used DNA, protein, or a combination of DNA and protein using a classical prime/boost strategy or delivered in a co-immunization regimen by combining the benefits of the 2 vaccine components. Magnitude, breadth, longevity and mucosal dissemination of anti-Env humoral responses were monitored in vaccinated macaques. We have previously shown that vaccination with DNA and protein in the form of AT-2 inactivated SIV virus particles leads to delayed virus acquisition after low dose repeated challenge with SIVsmE660 swarm [24] indicating that protection from infection may be achieved in the absence of a virus vector vaccine component. In this report, we examined the immune responses developed by such vaccine regimens, and we also explored the immunogenicity of DNA together with purified SIV Env protein instead of virus particles. We found that the systemic humoral responses (bAb and NAb) developed by the DNA&protein immunization are significantly higher. These data are in agreement with our recently reported pilot study in macaques, which received HIV env DNA and recombinant HIV gp120 protein [34]. Thus, co-immunization of HIV/SIV DNA and protein elicited not only the highest antibody responses in mice [33], [34], and rabbits [33] but also in macaques [[34] and this study]. Interestingly, inclusion of a protein with the DNA vaccine is beneficial for DNA delivered by needle/syringe [28], [29] as well as via electroporation ([24], [34] and herein). Our findings show that co-immunization of DNA&Protein provides a simplified vaccine regimen that increases immunization efficiency. Our work demonstrates that the combination of IM/EP delivery and the use of optimized DNA vectors achieve robust, durable immunity in macaques. It is possible that the nature of the immunogen produced endogenously by this method (proteins synthesized by the host with a perfect glycosylation pattern and all the natural posttranslational modifications; the types of cells that present the immunogen upon IM/EP) results in induction of more efficient immune responses. Future studies aim to delineate the underlying mechanisms. Importantly, the co-immunization regimen induces humoral responses of superior magnitude without compromising cellular immunity.

Moreover, higher systemic humoral responses also led to better dissemination of the anti-Env Ab responses to mucosal sites, such as the oral cavity and rectum. Importantly, from this cohort of macaques we had reported [24] that protection from SIVsmE660 infection correlated with plasma Env-specific bAb titers and the presence of rectal IgG to the heterologous challenge virus. In addition to delay in infection due to potent humoral responses, the study also showed that the vaccine regimens elicited effective cellular responses resulting in long-term control of viremia, with the majority (73%) of the vaccinated animals suppressing viremia to undetectable levels during the chronic phase of infection. Importantly, virus control was sustained during the entire study (40 weeks) resulting in complete prevention of CD4 depletion and any other signs of disease development in all the vaccinated animals. Thus, this reinforces our conclusion that these improved vaccine regimens led to the induction of immune responses of increased magnitude and breadth, better mucosal dissemination, and were able to protect against both infection and disease development.

A critical feature of any vaccine is the longevity of the induced humoral responses. The RV144 vaccine trial [25] elicited immune responses that waned rapidly after the vaccination period, suggesting that the transient nature of the elicited immunity was, at least partially, responsible for the limited vaccine efficacy. Those results indicate that improved vaccine designs are needed to achieve long-lasting, cross-clade specific immune responses able to prevent infection. In this report, we compared different vaccine regimens including DNA, protein and combination thereof and found that inclusion of DNA is critical for inducing long-lasting immunity. Although the protein only or the classical DNA prime–protein boost regimens induced responses of remarkable magnitude, the elicited humoral responses declined faster than those induced by regimens that included DNA (DNA only; DNA-protein co-immunization). These findings demonstrate that inclusion of DNA in the vaccine is critical to improve the longevity of responses. We report herein the maintenance of Env bAb in macaques for more than 3 years after the last vaccination. These data are corroborated by our previous report that SIV DNA only vaccination with IM/EP delivery is able to induce long-lasting (2 years) immune responses to Gag and Nef in macaques [23], which were boosted with each subsequent immunization, even after an extended 90-week rest period, indicating long-lasting memory responses. In a follow-up of some of these animals, we found an impressive durability of the Gag responses for >5 years. The remarkable longevity of the immune responses elicited by EP-delivered DNA vaccines extends data from another study with 1-year follow-up showing persistence of Env humoral responses in EP DNA vaccinated macaques [36]. The co-immunization regimen benefits from the inclusion of both vaccine components, eliciting both long-lasting humoral and cellular immune responses. Thus, the immunity induced by the co-immunization regimen is distinct from that obtained with replicating viral vectors like recombinant CMV, which provides robust, long-lasting SIV-specific CTL without inducing SIV-specific antibodies [37], [38]. Both recombinant CMV as well as DNA-based vaccines induced potent CTL responses able to efficiently control viremia for long periods of time [22], [24], [37]. However, in contrast to recombinant CMV vector, the DNA&Protein co-immunization also induces potent long-lasting systemic humoral immunity able to disseminate into mucosal surfaces and significantly reduce virus acquisition [24].

Together, our data show that a vaccine protocol combining DNA and protein elicits the most potent responses in macaques, characterized by persistent and efficient cellular immune responses [24] and the highest levels of functional humoral immune responses with remarkable longevity. Such properties may prove essential for the improvement of AIDS vaccines. Thus, this study supports a role of DNA and protein based vaccines for development of an efficacious HIV/AIDS vaccine.

## Materials and Methods

### Ethics Statement

This study was carried out in accordance with the Guide for the Care and Use of Laboratory Animals of the National Institutes of Health. Rhesus macaques were housed and handled in accordance with the standards of the Association for the Assessment and Accreditation of Laboratory Animal Care International at the Advanced BioScience Laboratories Inc., and were approved by the Institutional Animal Care and Use Committee (OLAW assurance number A3467-01 and USDA Certificate number 51-R-0059).

### Animal Cohorts & Vaccination

The animals in this study were Indian rhesus macaques (*Macaca mulatta*) and were housed at the Advanced BioScience Laboratories, Inc. (ABL) animal facility. All animals were cared for and procedures performed under a protocol approved by the ABL Animal Care and Use Committee (animal welfare assurance no. A3467-01; protocols AUP417, AUP490 and AUP516). The macaques in this study were managed according to the animal husbandry program of the ABL Animal Facility, which aims at providing consistent and excellent care to nonhuman primates at the vivarium. This program operates based on the laws, regulations, and guidelines promulgated by the United States Department of Agriculture (e.g., the Animal Welfare Act and its regulations, and the Animal Care Policy Manual), Institute for Laboratory Animal Research (e.g., Guide for the Care and Use of Laboratory Animals, 8th edition), Public Health Service, National Research Council, Centers for Disease Control, and the Association for Assessment and Accreditation of Laboratory Animal Care (AAALAC) International.

The nutritional plan utilized by the ABL Animal Facility consisted of twice daily feeding of Labdiet 5045 High Protein Primate Diet and food intake was closely monitored by Animal Research Technicians. This diet was also supplemented with a variety of fruits, vegetables, and other edible objects as part of the environmental enrichment program established by the Veterinary staff and enrichment Technician. Pairing of animals as part of the environmental enrichment program was managed by the enrichment technician. All primary enclosures and animal rooms were cleaned daily with water and sanitized at least once every two weeks. Seventy-five percent of the macaques used in this study were males. The animals had a weight range of 4–12 kg and a median age of 6 years with range from 3–19 years. Vaccinations were performed under anesthesia (Ketamine administered at 10 mg/kg) and all efforts were made to minimize suffering. None of the animals were euthanized as part of this study.

Ten macaques received two SIVmac239 env and gag DNA vaccinations as described [39], administered intramuscularly by needle and syringe at 0 and 2 months. Two additional macaques (33750, 35063) received the same DNA vaccine co-immunized with unadjuvanted aldrithiol-2 (AT-2) inactivated SIVmac239 particles [40] particles administered by subcutaneous and intramuscular injections into to same sites immediately following the DNA injection.

Groups of 8 macaques [20], [24] were vaccinated with a mixture of SIVmac239 env and gag DNA injected into the left and right inner thighs followed by EP using the ELGEN adaptive constant-current electroporator (Inovio Pharmaceuticals, Inc., Blue Bell, PA) as reported previously. The DNA vaccine mixture included rmIL-12 DNA, except for the DNA-only vaccinated animals shown in Figure 10. The animals received 4 vaccinations at weeks 0, 8, 16 and 36. Protein vaccination consisted of (AT-2) SIVmac239 particles (∼10 µg Env and 40 µg p27^gag^). The protein was injected intramuscularly and intradermally at the same sites as the DNA, following the DNA electroporation [24]. Two additional macaques (M437, P314) received 3 vaccinations (0, 2, 4 months) with the same amount of AT-2-inactivated particles together with sham DNA. Note, a different AT-2 inactivated particle preparation was used for macaques (33750, 35063) described above.

Two additional groups of macaques (N = 4 each) received a mixture of SIV gag DNA and Env gp160 DNA (SIVmac251_M766 [41], [42] and SIVsmE660_CG7V [41] gp140) and rmIL-12 DNA in four 0.25 ml injections (left and right internal thighs and upper arms) by IM/EP. One group of animals was co-immunized with 0.1 mg of matching recombinant gp140 Env proteins, purified from HEK293 cells [42], and formulated in 10 µg EM-005, an oil-in-water emulsion containing a TLR-4 agonist [35], as adjuvant in 200 µl PBS and injected by conventional IM injection at the same 4 sites as DNA, following the electroporation.

### Humoral Immune Responses

SIV-specific humoral immune responses were measured as detailed elsewhere [24], [42], [43]. Binding antibodies to SIVmac251 Env (Advanced BioScience Laboratories, Inc.) were measured by standard ELISA using duplicates of serially diluted plasma samples. End-point titers were defined as dilutions giving an OD higher than the cut-off value of the mean plus 3 times the standard deviation (SD) obtained from control plasma samples. Antibody titers to SIVmac239 and SIVsmE660 Env and antibody avidity of mac239 responses upon treatment with 1.5 M sodium thiocyanate (NaSCN; Sigma-Aldrich) were determined as described [44], [45]. The avidity index (%) was calculated by taking the ratio of the NaSCN-treated plasma dilution giving an absorbance of 0.5 to the TBS treated plasma dilution giving an OD of 0.5 and multiplying by 100. Neutralizing antibody titers were determined using the M7-luc assay for the TCLA-SIVmac251/H9 and the TZM-bl assay for SIVmac251_M766 and SIVsmE660_CG7G [46].

The SIV Env-specific IgG antibodies in plasma, saliva and rectal mucosa were determined by custom SIV bAb multiplex assay (SIV-BAMA) as previously described [24], [43], [47], [48]. Mucosal specimens were filtered and concentrated to equal volumes before measurement of total and specific antibody. Specific activity was calculated by the ratio of MFI (linear range of standard curve)/µg/ml total macaque IgG, which was measured by macaque IgG ELISA to normalize the data to recovery of antibody. The Weck cell sponges were examined for blood contamination and measured for semi quantitative evaluation of hemoglobin. Purified IgG from a SIV-infected macaque (DBM5, a gift from M. Roederer) was used as the positive control to calculate SIV antibody concentration. Positive controls for each antigen were tracked via Levy Jennings Plot. Specific activity was calculated from the total macaque IgG levels and the SIV specific concentrations. Antibodies against native V1/V2 epitopes were quantitated by binding assays against native SIV V1/V2 antigens expressed as gp70-fusion proteins related to the CaseA2 antigen used in the RV144 correlate study [26], [27]. These proteins contained the glycosylated, disulfide-bonded V1/V2 regions of SIVmac239, SIVmac251 and SIVsmE660 (corresponding to AA 120-204 of HXB2 Env), fused to residue 263 of the SU (gp70) protein of Fr-MuLV.

### Cellular Immune Responses

Cellular immune responses were measured by intracellular cytokine staining assay using cryopreserved PBMCs stimulated with Env peptide pools (15-mer overlapping by 11 AA at a final concentration of 1 µg/ml). Immunostaining and flow cytometric analysis was performed as described [22]–[24]. These data were internally controlled because every sample was run in the absence and in the presence of the Env-specific peptide pool. Only peptide-stimulated samples giving a frequency of IFN-γ^+^ T cells at least 2-fold higher than the negative controls (same samples without peptide stimulation) were considered positive. The frequency of IFN-γ^+^ T cells was reported after subtracting the background value obtained in the absence of peptides from that of peptide-stimulated samples.

### Statistical Analysis

Statistical analyses were performed using GraphPad Software, Inc. (version 6.0). P values have not been corrected for multiple comparisons.

## Supporting Information

Figure S1 **DNA&Protein co-immunization induces higher plasma Env antibodies measured by SIV bAb antibody multiplex assay (SIV-BAMA**)**.** bAb to SIVmac251 gp130 (A) and heterologous SIVsmE660 gp140 (B) were measured from plasma samples collected at 2 weeks post V4 using SIV-BAMA and are shown as mean fluorescent intensity (MFI). Asterisk (*) denotes that samples from 2 animals were not available. Median values are indicated. P values using non-parametric two-tailed t-test (Mann-Whitney) are shown.
(PDF)

Figure S2 **DNA and DNA&Protein co-immunization regimens induce similar Env-specific cellular immune responses.** Env-specific cellular immune responses were measured from PBMC collected as described in Figure 3. (A) Comparison of the Env-specific IFN-γ+ T cell responses in PBMC collected at 2 weeks post V2 from the groups that received 2 vaccinations with DNA only (N = 16) and DNA&Protein co-immunization (N = 8), respectively. Median values are indicated. P values using non-parametric two-tailed t-test (Mann-Whitney) are shown. (B) The Env-specific CD4+ and CD8+ IFN-γ+ T cells responses of the individual animals shown in panel A are plotted. (C) Comparison of the Env-specific IFN-γ+ T cell responses after 4 vaccinations in the groups that received 4 DNA immunizations (N = 8), 4 DNA&Protein co-immunizations (N = 8) and 2 DNA prime followed by 2 protein boosts immunizations (N = 8), respectively. Note the response of the DNA prime-protein boost group at V4wk2 time point were measured 5.5 months post V2, the last time the animals received DNA. Median values are indicated. P values using non-parametric two-tailed t-test (Mann-Whitney) are shown.
(PDF)
