# Supplementary figures and images for: DNA and Protein Co-Immunization Improves the Magnitude and Longevity of Humoral Immune Responses in Macaques
Source: PLoS One. 2014 Mar 13;9(3):e91550. doi: 10.1371/journal.pone.0091550 (PMC3953433; doi:10.1371/journal.pone.0091550)

**A**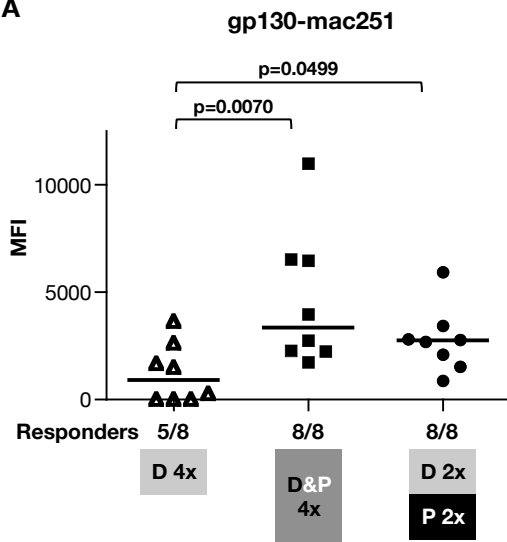**B**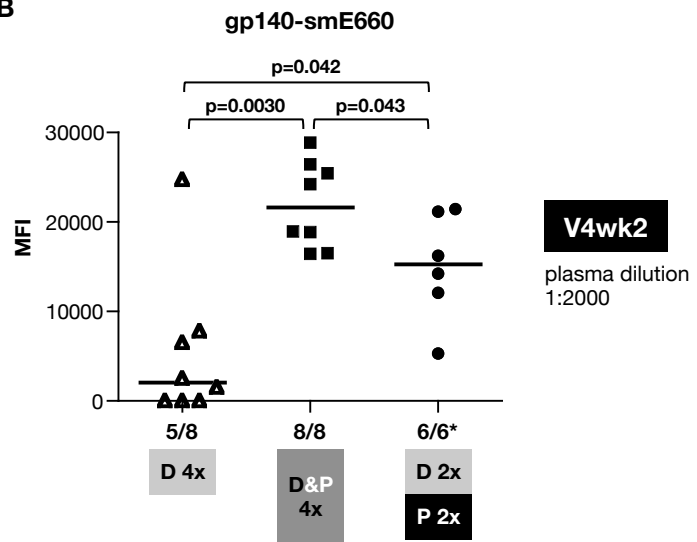

Supplement: Figure S1 — DNA&Protein co-immunization induces higher plasma Env antibodies measured by SIV bAb antibody multiplex assay (SIV-BAMA). bAb to SIVmac251 gp130 (A) and heterologous SIVsmE660 gp140 (B) were measured from plasma samples collected at 2 weeks post V4 using SIV-BAMA and are shown as mean fluorescent intensity (MFI). Asterisk (*) denotes that samples from 2 animals were not available. Median values are indicated. P values using non-parametric two-tailed t-test (Mann-Whitney) are shown. (PDF) [file pone.0091550.s001.pdf]

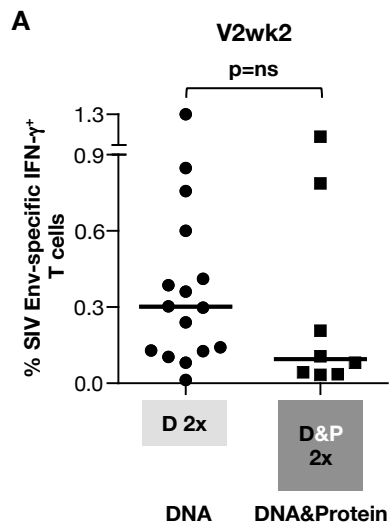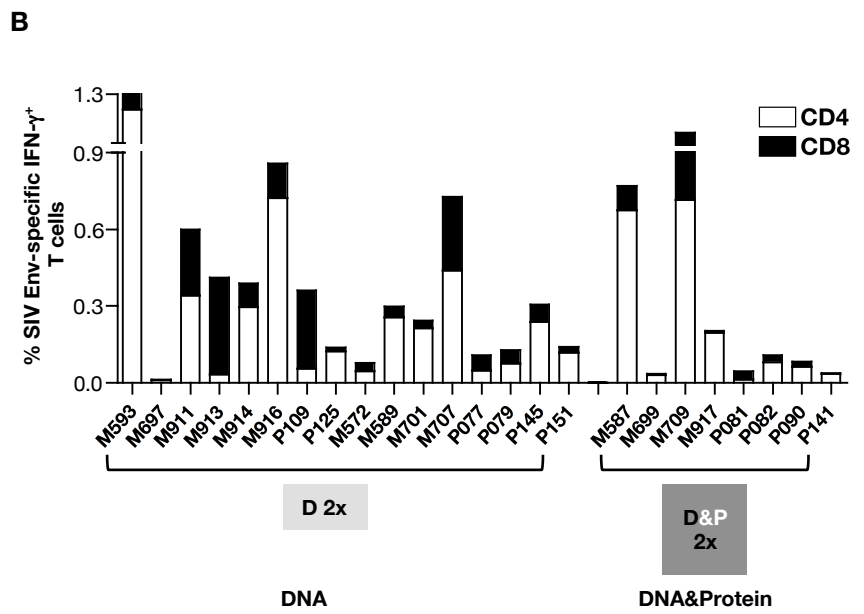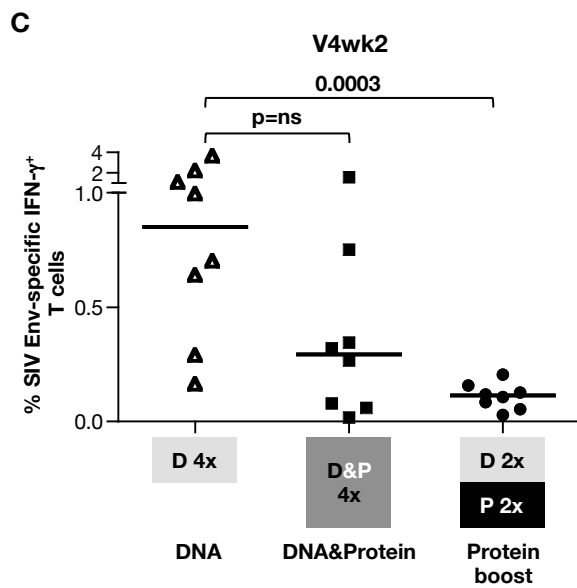

Supplement: Figure S2 — DNA and DNA&Protein co-immunization regimens induce similar Env-specific cellular immune responses. Env-specific cellular immune responses were measured from PBMC collected as described in Figure 3. (A) Comparison of the Env-specific IFN-γ+ T cell responses in PBMC collected at 2 weeks post V2 from the groups that received 2 vaccinations with DNA only (N = 16) and DNA&Protein co-immunization (N = 8), respectively. Median values are indicated. P values using non-parametric two-tailed t-test (Mann-Whitney) are shown. (B) The Env-specific CD4+ and CD8+ IFN-γ+ T cells responses of the individual animals shown in panel A are plotted. (C) Comparison of the Env-specific IFN-γ+ T cell responses after 4 vaccinations in the groups that received 4 DNA immunizations (N = 8), 4 DNA&Protein co-immunizations (N = 8) and 2 DNA prime followed by 2 protein boosts immunizations (N = 8), respectively. Note the response of the DNA prime-protein boost group at V4wk2 time point were measured 5.5 months post V2, the last time the animals received DNA. Median values are indicated. P values using non-parametric two-tailed t-test (Mann-Whitney) are shown. (PDF) [file pone.0091550.s002.pdf]
